# Association Between Any Circulating ANCA Positivity at Diagnosis and Subsequent End-Stage Kidney Disease in Microscopic Polyangiitis and Granulomatosis with Polyangiitis

**DOI:** 10.3390/medicina62091699

**Published:** 2026-09-04

**Authors:** Jang Woo Ha, Jeong Yeop Whang, Oh Chan Kwon, Yong-Beom Park, Sang-Won Lee

**Affiliations:** 1 Division of Rheumatology, Department of Internal Medicine, Yongin Severance Hospital, Yonsei University College of Medicine, Yongin 16995, Republic of Korea; hjwnmk@yuhs.ac; 2 Department of Medicine, Yonsei University College of Medicine, Seoul 03722, Republic of Korea; 3 Division of Rheumatology, Department of Internal Medicine, Gangnam Severance Hospital, Yonsei University College of Medicine, Seoul 06273, Republic of Korea; ockwon@yuhs.ac; 4 Division of Rheumatology, Department of Internal Medicine, Yonsei University College of Medicine, Seoul 03722, Republic of Korea; yongbpark@yuhs.ac; 5 Institute for Immunology and Immunological Diseases, Yonsei University College of Medicine, Seoul 03722, Republic of Korea

**Keywords:** microscopic polyangiitis, granulomatosis with polyangiitis, antineutrophil cytoplasmic antibody, positivity, end-stage kidney disease

## Abstract

*Background and Objectives*: This study examined whether positivity for any antineutrophil cytoplasmic antibody (ANCA) at diagnosis was associated with subsequent advanced systemic complications during follow-up in patients with microscopic polyangiitis (MPA) and granulomatosis with polyangiitis (GPA). *Materials and Methods*: We retrospectively reviewed the medical records of 271 patients with MPA or GPA enrolled in the ANCA-associated vasculitis cohort at a tertiary hospital. Any ANCA positivity was defined as the presence of any of the following: myeloperoxidase-ANCA, proteinase 3-ANCA, perinuclear ANCA, or cytoplasmic ANCA. Subsequent advanced systemic complications during follow-up, such as all-cause mortality and end-stage kidney disease (ESKD), were evaluated. *Results*: The median age of the 271 patients with MPA or GPA was 62.0 years, and 239 and 32 were identified as ANCA-positive and ANCA-negative vasculitis, respectively. During follow-up, 47 patients (17.3%) died, and 52 (19.2%) progressed to ESKD. In a cross-sectional comparative analysis, ESKD occurred significantly more frequently in ANCA-positive patients than in ANCA-negative patients. Among the five subsequent advanced systemic complications of MPA and GPA, ANCA-negative patients exhibited a significantly higher cumulative ESKD-free survival rate than ANCA-positive patients. However, in multivariable Cox proportional hazards analysis adjusted for age, sex, AAV subtype, and baseline serum creatinine, ANCA positivity was not independently associated with subsequent ESKD. *Conclusions*: Any ANCA positivity at diagnosis was associated with subsequent ESKD in unadjusted analyses but was not an independent predictor after adjustment for baseline renal function and other clinically relevant covariates.

## 1. Introduction

Antineutrophil cytoplasmic antibody (ANCA)-associated vasculitis (AAV) is a group of small-vessel vasculitides characterized by fibrinoid necrosis and few or no immune-complex depositions. Microscopic polyangiitis (MPA) and granulomatosis with polyangiitis (GPA) belong to this disease group but are distinguished from each other by their clinical and histopathological characteristics [1,2]. Serologically, MPA and GPA show subtype-specific ANCAs and are associated with myeloperoxidase (MPO)-ANCA and proteinase 3 (PR3)-ANCA, respectively, in their pathogenesis [3,4,5]. In the 2022 American College of Rheumatology (ACR) and the European Alliance of Associations for Rheumatology (EULAR) classification criteria for MPA and GPA, ANCA findings concordant with each disease are given substantial positive weights, whereas discordant ANCA findings receive negative scores [6,7,8]. Therefore, although circulating ANCAs and their titres do not reflect the current activity of the disease, MPO-ANCA and PR3-ANCA have been recognised as key players in classifying or diagnosing MPA and GPA, respectively, at the time of diagnosis.

Given the role of circulating ANCA in the pathogenesis of MPA and GPA, it can be reasonably inferred that not only the vasculitic activity, extent of inflammation, and degree of organ damage at diagnosis but also subsequent advanced systemic complications during follow-up may differ depending on the presence or absence of ANCA, irrespective of the ANCA type [9,10]. However, in clinical practice, we encounter heterogeneous situations, such as patients with an ambiguous borderline between MPA and GPA at diagnosis; those with MPO-ANCA positivity at diagnosis but classified as having GPA; those with PR3-ANCA positivity at diagnosis but classified as having MPA; and those positive for both ANCAs or with no ANCA at diagnosis but classified as MPA or GPA. In addition to these situations, considering that the algorithm for AAV suggested by the European Medicines Agency (EMA) in 2007 included ‘any ANCA positivity’ rather than ‘MPO-ANCA or PR3-ANCA positivity’ [2,8], it was believed to be more practical to assess the relationship between any ANCA positivity at diagnosis and subsequent advanced systemic complications during follow-up. Previous studies examining the clinical implications of ANCA status have largely focused on biopsy-proven pauci-immune glomerulonephritis, renal-limited vasculitis, antigen-specific MPO-ANCA and PR3-ANCA phenotypes, or longitudinal changes in ANCA titres. In contrast, the clinical implications of a pragmatic binary measure of any detectable circulating ANCA in patients classified as having MPA or GPA remain less well characterized. Therefore, this study investigated whether any ANCA positivity at diagnosis, irrespective of antigen specificity or immunofluorescence pattern, was associated with subsequent advanced systemic complications during follow-up in patients with MPA and GPA classified according to the 2022 ACR/EULAR criteria.

## 2. Materials and Methods

### 2.1. Patient Selection

The inclusion criteria were as follows: (i) patients whose initial classification as having MPA or GPA was made at this tertiary hospital; (ii) newly diagnosed patients meeting the 2022 ACR/EULAR classification criteria for MPA or GPA [6,7,8]; (iii) patients previously classified as having MPA or GPA who satisfied the corresponding 2022 ACR/EULAR criteria [1,2,6,7]; (iv) ANCA test results at diagnosis based on the detection methods [11,12]; (v) well-written medical records containing the contents of clinical data at diagnosis as well as those during follow-up, in particular, those regarding advanced systemic complications of MPA and GPA; (vi) absence of concurrent medical conditions that may mimic MPA and GPA at diagnosis, such as malignancies and serious infectious and/or metabolic diseases [13,14]; (vii) no exposure to immunosuppressive drugs for treating MPA or GPA, such as rituximab, cyclophosphamide, azathioprine, mycophenolate mofetil, calcineurin inhibitors, or methotrexate, within 4 weeks before diagnosis [15,16]; (viii) no exposure to glucocorticoids (prednisolone ≥ 10 mg) within 4 weeks before diagnosis; and (ix) follow-up duration of >6 months after diagnosis. Based on these criteria, 336 patients from the AAV cohort at a tertiary hospital were screened. After screening 336 individuals, 271 patients classified as having MPA or GPA satisfied all eligibility requirements and formed the final study population. Data were obtained through a retrospective review of their medical records. The study protocol received approval from the Institutional Review Board of Severance Hospital in Seoul, Republic of Korea, on 5 December 2016 (IRB No. 4-2016-0901), and all study procedures complied with the Declaration of Helsinki. Because the analysis was retrospective and used de-identified clinical information, the Institutional Review Board waived the requirement to obtain written informed consent.

### 2.2. Clinical Data at the Time of Diagnosis

At diagnosis, age, sex, body mass index, and smoking history were recorded as demographic characteristics, together with the presence of type 2 diabetes mellitus, hypertension, and dyslipidaemia [17]. We also collected AAV subtype and ANCA status and assessed disease-related indices, including the Birmingham Vasculitis Activity Score (BVAS) version 3, the Five-Factor Score (FFS), and the 36-item Short-Form Survey Physical and Mental Component Summary (SF-36 PCS and MCS) [18,19,20]. Major organ involvement was also evaluated, based on the nine systemic items of BVAS [18]. Laboratory data obtained at diagnosis included erythrocyte sedimentation rate (ESR) and C-reactive protein (CRP) as measures of the acute-phase response [21].

### 2.3. Clinical Data During Follow-Up

During follow-up, we evaluated five prespecified outcomes: all-cause mortality (ACM), relapse, end-stage kidney disease (ESKD), cerebrovascular accident (CVA), and acute coronary syndrome (ACS). ACM and relapse were defined as death due to any cause and recurrence after a remission, respectively. ESKD was defined as kidney failure requiring maintenance dialysis for at least 90 consecutive days after the diagnosis of MPA or GPA [22]. Stroke was categorized as CVA, while ACS encompassed ST-elevation and non-ST-elevation myocardial infarction as well as unstable angina [23,24]. For each outcome, follow-up time was calculated from the date of diagnosis to the first occurrence of that outcome or, for patients without the event, to their last documented follow-up. We additionally reviewed the use of glucocorticoids, rituximab, cyclophosphamide, mycophenolate mofetil, azathioprine, tacrolimus, and methotrexate during follow-up.

### 2.4. ANCA Measurements and Accepted Values

ANCA status was determined using two complementary techniques. An enzyme immunoassay quantified the titres of MPO-ANCA and PR3-ANCA, whereas an indirect immunofluorescent assay identified perinuclear (P)-ANCA and cytoplasmic (C)-ANCA patterns [11,12]. Based on the 2022 ACR/EULAR classification criteria for MPA and GPA, the results of both assays were considered valid in the present study [6,7]. ‘Any ANCA positivity’ or ‘ANCA-positive’ was defined as a case in which at least one of the four ANCA test results, including MPO-ANCA, PR3-ANCA, P-ANCA, and C-ANCA, was positive. ANCA positivity was determined according to the laboratory-specific reference range in effect at the time of testing because assay platforms and reference ranges changed during the study period.

### 2.5. Statistical Analyses

All analyses were conducted using IBM SPSS Statistics for Windows, version 26.0 (IBM Corp., Armonk, NY, USA). Continuous variables are presented as medians with interquartile ranges, while categorical variables are shown as numbers and percentages. Categorical variables were compared between groups using Pearson’s chi-square test or Fisher’s exact test, as appropriate, and continuous variables were analysed using the Mann–Whitney U test. Kaplan–Meier curves were constructed for time-to-event outcomes and compared using the log-rank test. To examine whether ANCA positivity was independently associated with subsequent ESKD, Cox proportional hazards regression was performed with adjustment for age, sex, AAV subtype, and baseline serum creatinine. The proportional hazards assumption was assessed using time-dependent covariates generated by interactions between each variable included in the Cox model and log-transformed follow-up time. Effect estimates are reported as hazard ratios (HRs) with 95% confidence intervals (CIs). All statistical tests were two-sided, with *p* < 0.05 considered statistically significant.

## 3. Results

### 3.1. Clinical Data at Diagnosis

The median age of the 271 patients with MPA or GPA was 62.0 years, and 99 and 172 were men and women, respectively. Among the patients, 190 and 81 were diagnosed with MPA and GPA, respectively, and 239 and 32 were identified as ANCA-positive and ANCA-negative patients with MPA or GPA, respectively, as well. Of the 271 patients, 204 showed MPO-ANCA (or P-ANCA) positivity, and 45 showed PR3-ANCA (or C-ANCA) positivity. Median values were 12.0 for BVAS, 1.0 for FFS, 48.8 for SF-36 PCS, and 54.1 for SF-36 MCS. Kidney and lung involvement were observed in 67.2% and 63.8% of patients, respectively, and represented the two most frequent major organ manifestations. Laboratory results are presented in Table 1.

### 3.2. Clinical Data During Follow-Up

Among the 271 patients with MPA or GPA, 47 (17.3%) died during a median follow-up of 56.3 months.

During follow-up, relapse occurred in 73 patients (26.9%), ESKD in 52 (19.2%), CVA in 18 (6.6%), and ACS in 7 (2.6%). Nearly all patients received glucocorticoids (96.7%). Among the immunosuppressive agents, cyclophosphamide was used most commonly (52.8%), followed by azathioprine (46.5%) and mycophenolate mofetil (28.4%) (Table 2).

### 3.3. Comparison Analysis of Clinical Data

Among the clinical variables at diagnosis, ANCA-positive patients were older than ANCA-negative patients (64 vs. 56 years, *p* = 0.001). ANCA-positive patients also exhibited a significantly higher BVAS (12.0 vs. 9.5, *p* = 0.025) and FFS (2.0 vs. 1.0, *p* = 0.002) than ANCA-negative patients. Additionally, ANCA-positive patients had significantly higher platelet count (291,000/mm^3^ vs. 277,500/mm^3^, *p* = 0.013), blood urea nitrogen (20.1 mg/dL vs. 16.3 mg/dL, *p* = 0.002), serum creatinine (1.0 mg/dL vs. 0.7 mg/dL, *p* = 0.003), ESR (67.0 mm/h vs. 39.5 mm/h, *p* < 0.001, and CRP levels (13.6 mg/L vs. 1.8 mg/L, *p* = 0.001) but significantly lower white blood cell counts (8725/mm^3^ vs. 9660/mm^3^, *p* = 0.013), haemoglobin levels (10.9 g/dL vs. 11.7 g/dL, *p* = 0.003), and serum albumin levels (3.7 g/dL vs. 3.9 g/dL, *p* = 0.007) than ANCA-negative patients (Table 3).

Follow-up periods for all assessed systemic outcomes were significantly shorter in the ANCA-positive group than in the ANCA-negative group. Progression to ESKD was observed in 20.9% of ANCA-positive patients compared with 6.3% of ANCA-negative patients (*p* = 0.048). In contrast, the frequencies of ACM, relapse, CVA, and ACS were comparable between the groups. Among medications, rituximab (25.1% vs. 9.4%, *p* = 0.048) and mycophenolate mofetil (31.0% vs. 9.4%, *p* = 0.011) were more frequently administered in the ANCA-positive group than in the ANCA-negative group. Conversely, ANCA-positive patients received methotrexate less frequently than ANCA-negative patients (13.4% vs. 37.5%, *p* < 0.001) (Table 3).

### 3.4. Comparison of Systemic Manifestations and Poor Outcomes According to Abnormal ANCA Positivity

**Figure 1:**
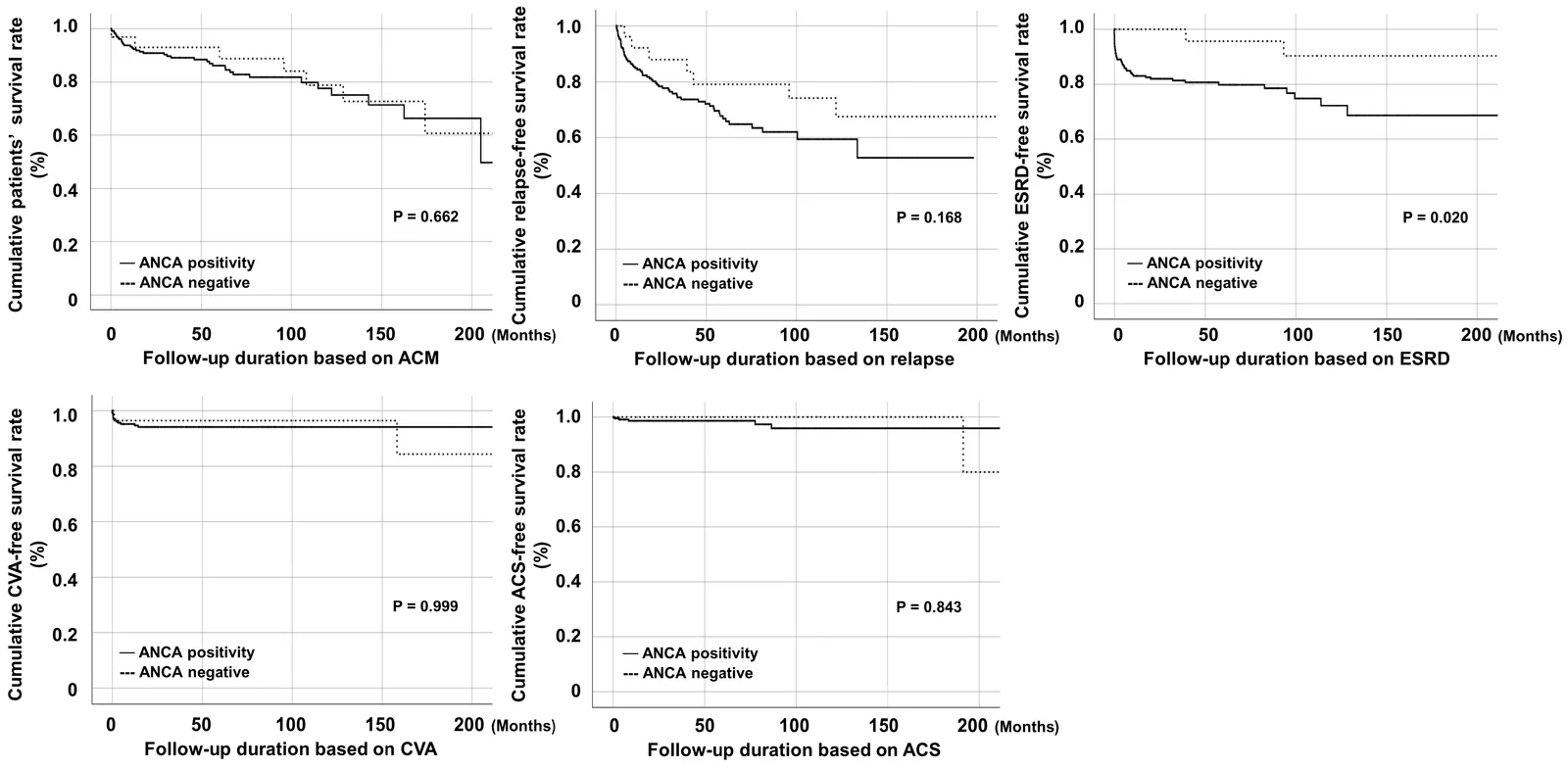
Comparison of cumulative survival rates. ANCA-negative patients exhibited a significantly higher cumulative ESKD-free survival rate than ANCA-positive patients during follow-up. ANCA: antineutrophil cytoplasmic antibody; ESKD: end-stage kidney disease.

Among the five subsequent advanced systemic complications of MPA and GPA, ANCA-negative patients exhibited a significantly higher cumulative ESKD-free survival rate than ANCA-positive patients during follow-up (*p* = 0.020). However, the remaining advanced systemic complications showed no significant differences in cumulative survival rates between the two groups based on the presence of ANCA (Figure 1).

In multivariable Cox proportional hazards analysis adjusted for age, sex, AAV subtype, and baseline serum creatinine, ANCA positivity was not independently associated with subsequent ESKD (HR 2.764, 95% CI 0.651–11.740, *p* = 0.168). In contrast, older age (HR 1.035, 95% CI 1.008–1.062, *p* = 0.009) and higher baseline serum creatinine (HR 1.589, 95% CI 1.453–1.738, *p* < 0.001) were independently associated with subsequent ESKD. No significant time-dependent interaction was observed for any variable included in the model, indicating no evidence of violation of the proportional hazards assumption. The complete results of the multivariable Cox proportional hazards analysis are presented in Table 4.

## 4. Discussion

Given the role of circulating ANCA in the pathogenesis of AAV, and further, the clinical situations showing the ambiguous consistency of MPO-ANCA and PR3-ANCA with ANCA subtypes, we considered it more practical to assess the relationship between any ANCA positivity detected at diagnosis and subsequent advanced systemic complications of MPA and GPA during follow-up. Hence, the present study investigated whether any ANCA positivity at diagnosis was associated with subsequent advanced systemic complications after diagnosis in patients with MPA and GPA. Several interesting findings were obtained. First, among the 271 patients with MPA or GPA, 239 and 32 were identified as ANCA-positive and ANCA-negative patients with MPA or GPA, respectively. Second, among the 271 patients with MPA or GPA, 47 (17.3%) died during a median follow-up of 56.3 months, and 73 (26.9%), 52 (19.2%), 18 (6.6%), and 7 (2.6%) had subsequent relapse, ESKD, CVA, and ACS, respectively, during follow-up. Third, in the comparative analyses of the variables at diagnosis, ANCA-positive patients exhibited significantly higher BVAS, FFS, platelet count, blood urea nitrogen, serum creatinine, ESR, and CRP levels, and conversely, significantly lower white blood cell counts, haemoglobin levels, and serum albumin levels than ANCA-negative patients. Fourth, in the comparison of outcomes during follow-up, ESKD occurred significantly more frequently in ANCA-positive patients than in ANCA-negative patients, whereas no significant differences were observed in ACM, relapse, CVA, or ACS between the two groups. Fifth, among the five subsequent advanced systemic complications of MPA and GPA, ANCA-negative patients exhibited a significantly higher cumulative ESKD-free survival rate than ANCA-positive patients during follow-up.

Although ANCA-positive patients exhibited a higher frequency of ESKD and lower cumulative ESKD-free survival than ANCA-negative patients in the unadjusted analyses, ANCA positivity was not independently associated with subsequent ESKD after adjustment for age, sex, AAV subtype, and baseline serum creatinine (HR 2.764, 95% CI 0.651–11.740, *p* = 0.168). In contrast, higher baseline serum creatinine remained independently associated with subsequent ESKD (HR 1.589, 95% CI 1.453–1.738, *p* < 0.001). These findings suggest that the observed association between ANCA positivity and subsequent ESKD may, at least in part, reflect differences in the baseline clinical phenotype, particularly the extent of renal involvement and renal dysfunction, rather than an independent prognostic effect of ANCA positivity itself. Indeed, ANCA-positive patients in the present study had more frequent renal involvement and higher serum creatinine levels at diagnosis than ANCA-negative patients. Therefore, any ANCA positivity at diagnosis may serve as a clinical marker identifying patients with a greater baseline renal disease burden but should not be interpreted as an independent predictor of subsequent ESKD.

In a simple comparison, the incidence of ESKD was significantly higher in ANCA-positive patients than in ANCA-negative patients. The survival analysis demonstrated a significantly lower cumulative ESKD-free survival in ANCA-positive patients than in ANCA-negative patients. The first hypothesis is that any ANCA positivity may directly be associated with renal damage and functional impairment secondary to kidney involvement, which ultimately may serve as the initiator for subsequent progression to ESKD [4,5,9,10]. ANCA-positive patients showed elevated blood urea nitrogen and serum creatinine levels at diagnosis compared to ANCA-negative patients. Although this does not provide evidence of a detailed link between ANCA positivity and elevated serum creatinine, it may suggest an association between ANCA positivity and the cross-sectional extent of renal injury [18,19]. The second hypothesis is that any ANCA positivity may indirectly be associated with renal damage and functional impairment secondary to systemic inflammatory conditions rather than direct kidney involvement, which may contribute to subsequent progression to ESKD [25]. The finding that ANCA-positive patients showed significantly higher ESR and CRP levels compared to ANCA-negative patients may support this hypothesis, and further, the significantly higher FFS assessed in ANCA-positive patients at diagnosis also may serve as another line of evidence supporting this hypothesis [26,27]. These observations raise the possibility that the presence of any ANCA at diagnosis, independent of its type, may directly reflect the potential for concurrent renal injury (cross-sectional damage) and can indirectly mirror the current systemic inflammatory status that may induce renal damage and functional deterioration either concurrently or in the near future.

Considering that initial impaired renal function was the most critical factor influencing the subsequent progression to ESKD [28,29], we performed an exploratory subgroup analysis restricted to patients without renal insufficiency at diagnosis, defined as a serum creatinine level < 1.7 mg/dL based on the renal insufficiency item of the FFS [19]. Of the 271 patients, 192 with serum creatinine < 1.7 mg/dL at the time of diagnosis were included in this subgroup analysis. Of the 192 patients, 128 and 64 were diagnosed with MPA and GPA, and 15.1% were classified as having ANCA-negative MPA or GPA. When comparing the two groups, no significant difference in serum creatinine levels was observed between ANCA-positive and ANCA-negative patients (0.8 mg/dL vs. 0.7 mg/dL, *p* = 0.154). By contrast, ANCA-positive patients still exhibited significantly elevated ESR and CRP levels compared to ANCA-negative patients (68.0 mm/h vs. 39.5 mm/h, *p* < 0.001, and 13.2 mg/L vs. 1.8 mg/L, *p* = 0.002). However, cumulative ESKD-free survival did not differ significantly between ANCA-positive and ANCA-negative patients in this subgroup (*p* = 0.086) (Supplementary Figure S1), suggesting that the association observed in the overall cohort may have been influenced, at least in part, by baseline renal dysfunction.

The strength of this study lies in its simplified analysis of the clinical significance of circulating ANCA positivity in patients newly classified as having MPA or GPA and its evaluation of the association between any ANCA positivity and subsequent ESKD. Furthermore, this study has clinical implications in that it addresses the real-world clinical scenario in which the AAV subtype and its specific ANCA type do not always align. Another strength of this study is that it included a substantial number of patients with MPA and GPA who were enrolled and maintained according to well-established protocols. This study had several limitations. First, the single-centre design and relatively limited sample size may restrict the external validity of our findings, particularly their applicability to other populations of newly diagnosed patients. However, the disadvantage of a single-centre study was expected to be offset by the fact that the diagnosis and follow-up were conducted by the same medical team using the same but consistently updated criteria. Second, because this study was conducted retrospectively, the possibility of missing or uncertain information, compared to that in a prospective study, could not be overcome. Although time-to-event analyses accounted for unequal follow-up and censoring, residual calendar-time effects, treatment-era differences, and informative censoring cannot be completely excluded because of the retrospective study design. Third, another limitation was the lack of regularly and consecutively performed ANCA tests during the follow-up period after diagnosis, even when considering the potential influence of medications administered after diagnosis on serial ANCA results. Death was not formally modelled as a competing event for ESKD. Therefore, the Kaplan–Meier findings should be interpreted as conventional time-to-event comparisons rather than estimates of the absolute cumulative incidence of ESKD, particularly given the small number of ESKD events in the ANCA-negative group. Because ANCA results were retrospectively collected over an extended period during which assay platforms and laboratory reference ranges changed, uniform assay-specific cut-off values could not be reliably reconstructed. In addition, only two patients in the ANCA-negative group progressed to ESKD. Consequently, the adjusted effect estimate for ANCA positivity was imprecise, as reflected by the wide 95% CI (0.651–11.740), and the magnitude of this association should therefore be interpreted cautiously. Larger prospective investigations are warranted to address these limitations and to more clearly define the clinical implications of ANCA positivity at diagnosis throughout the disease course of MPA and GPA.

## 5. Conclusions

Any ANCA positivity at diagnosis was associated with a higher occurrence of ESKD and lower cumulative ESKD-free survival in unadjusted analyses. However, this association was attenuated after adjustment for baseline renal function and other clinically relevant covariates. These findings suggest that ANCA positivity may identify a clinical phenotype associated with greater renal risk rather than serving as an independent predictor of subsequent ESKD.

## Figures and Tables

**Table 1:** Clinical data at diagnosis in MPA and GPA patients (N = 271).

Variables	Values
Demographic data	
Age (years)	62.0 (51.0–71.0)
Male sex (N, (%))	99 (36.5)
Female sex (N, (%))	172 (63.5)
BMI (kg/m^2^)	22.5 (20.6–24.6)
Ex-smoker	7 (2.6)
Comorbidities (N, (%))	
T2DM	54 (19.9)
Hypertension	109 (40.2)
Dyslipidaemia	45 (16.6)
AAV subtype (N, (%))	
MPA	190 (70.1)
GPA	81 (29.9)
ANCA type and positivity (N, (%))	
ANCA positivity	239 (88.2)
ANCA negativity	32 (11.8)
MPO-ANCA (or P-ANCA) positivity	204 (75.3)
PR3-ANCA (or C-ANCA) positivity	45 (16.6)
Both ANCAs	10 (3.7)
AAV-specific indices	
BVAS	12.0 (6.0–18.0)
FFS	1.0 (1.0–2.0)
SF-36 PCS	48.8 (31.9–63.8)
SF-36 MCS	54.1 (37.8–70.3)
Major organ involvement based on BVAS items (N, (%))	
General manifestations	118 (43.5)
Cutaneous manifestations	40 (14.8)
Mucous/Ocular manifestations	19 (7.0)
Otorhinolaryngological manifestations	100 (36.9)
Pulmonary manifestations	173 (63.8)
Cardiovascular manifestations	50 (18.5)
Gastrointestinal manifestations	7 (2.6)
Renal manifestations	182 (67.2)
CNS/PNS manifestations	67 (24.7)
Laboratory results	
White blood cell count (/mm^3^)	8800.0 (6280.0–12,330.0)
Haemoglobin (g/dL)	11.0 (9.4–12.8)
Platelet count (×1000/mm^3^)	294.0 (225.0–393.0)
Fasting glucose (mg/dL)	100.0 (91.0–127.0)
Blood urea nitrogen (mg/dL)	18.8 (14.1–35.0)
Serum creatinine (mg/dL)	0.9 (0.7–2.0)
Serum total protein (g/dL)	6.8 (6.1–7.2)
Serum albumin (g/dL)	3.7 (3.1–4.2)
ESR (mm/h)	63.0 (24.0–102.0)
CRP (mg/L)	14.8 (1.8–68.2)

Values are expressed as a median (25–75 percentile) or N (%). MPA: microscopic polyangiitis; GPA: granulomatosis with polyangiitis; BMI: body mass index; T2DM: type 2 diabetes mellitus; MPO: myeloperoxidase; ANCA: antineutrophil cytoplasmic antibody; P: perinuclear; PR3: proteinase 3; C: cytoplasmic; BVAS: the Birmingham Vasculitis Activity Score; FFS: the Five-Factor Score; SF-36: 36-item Short-Form Survey; PCS: Physical Component Summary; MCS: Mental Component Summary; CNS: central nervous system; PNS: peripheral nervous system; ESR: erythrocyte sedimentation rate; CRP: C-reactive protein.

**Table 2:** Clinical data during follow-up in MPA and GPA patients (N = 271).

Variables	Values
Advanced systemic complications	
ACM (N, (%))	47 (17.3)
Follow-up duration based on ACM (months)	56.3 (19.9–99.6)
Relapse (N, (%))	73 (26.9)
Follow-up duration based on relapse (months)	31.8 (7.4–72.6)
ESKD (N, (%))	52 (19.2)
Follow-up duration based on ESKD (months)	43.1 (9.2–89.8)
CVA (N, (%))	18 (6.6)
Follow-up duration based on CVA (months)	51.9 (13.4–97.6)
ACS (N, (%))	7 (2.6)
Follow-up duration based on ACS (months)	54.7 (19.1–98.5)
Medications administered (N, (%))	
Glucocorticoid	262 (96.7)
Rituximab	62 (22.9)
Cyclophosphamide	143 (52.8)
Mycophenolate mofetil	77 (28.4)
Azathioprine	126 (46.5)
Tacrolimus	23 (8.5)
Methotrexate	44 (16.2)

Values are expressed as a median (25–75 percentile) or N (%). MPA: microscopic polyangiitis; GPA: granulomatosis with polyangiitis; ACM: all-cause mortality; ESKD: end-stage kidney disease; CVA: cerebrovascular accident; ACS: acute coronary syndrome.

**Table 3:** Comparative analysis between ANCA-positive and ANCA-negative patients with MPA or GPA.

Variables	ANCA-Positive Patients (N = 239)	ANCA-Negative Patients (N = 32)	*p*-Value
At diagnosis			
Demographic data			
Age (years)	64.0 (55.0–71.8)	56.0 (37.8–69.3)	0.001
Male sex (N, (%))	90 (37.7)	9 (28.1)	0.293
Female sex (N, (%))	149 (62.3)	23 (71.9)	0.293
BMI (kg/m^2^)	22.4 (20.5–24.3)	22.0 (18.8–24.3)	0.735
Ex-smoker	5 (2.1)	2 (6.3)	0.195
Comorbidities (N, (%))			
T2DM	48 (20.1)	6 (18.8)	0.859
Hypertension	101 (42.3)	8 (25.0)	0.062
Dyslipidaemia	41 (17.2)	4 (12.5)	0.506
AAV subtype (N, (%))			0.158
MPA	171 (71.5)	19 (59.4)	
GPA	68 (28.5)	13 (40.6)	
ANCA type and positivity (N, (%))			
MPO-ANCA (or P-ANCA) positivity	204 (85.4)	0 (0)	<0.001
PR3-ANCA (or C-ANCA) positivity	45 (18.8)	0 (0)	0.007
Both ANCAs	10 (4.2)	0 (0)	0.613
AAV-specific indices			
BVAS	12.0 (6.3–18.0)	9.5 (5.5–14.8)	0.025
FFS	2.0 (1.0–2.0)	1.0 (0.8–2.0)	0.002
SF-36 PCS	48.4 (30.3–63.6)	48.6 (39.4–74.2)	0.178
SF-36 MCS	54.2 (36.4–69.7)	57.7 (45.2–79.5)	0.121
Major organ involvement based on BVAS items (N, (%))			
General manifestations	108 (45.2)	10 (31.3)	0.135
Cutaneous manifestations	26 (10.9)	14 (43.8)	<0.001
Mucous/Ocular manifestations	14 (5.9)	5 (15.6)	0.058
Otorhinolaryngological manifestations	86 (36.0)	14 (43.8)	0.393
Pulmonary manifestations	164 (68.6)	9 (28.1)	<0.001
Cardiovascular manifestations	43 (18.0)	7 (21.9)	0.595
Gastrointestinal manifestations	6 (2.5)	1 (3.1)	0.589
Renal manifestations	166 (69.5)	16 (50.0)	0.028
CNS/PNS manifestations	57 (23.8)	10 (31.3)	0.362
Laboratory results			
White blood cell count (/mm^3^)	8725.0 (6295.0–12,395.0)	9660.0 (7122.5–13,955.0)	0.013
Haemoglobin (g/dL)	10.9 (9.3–12.7)	11.7 (10.3–13.3)	0.003
Platelet count (×1000/mm^3^)	291.0 (210.5–388.8)	277.5 (228.3–352.8)	0.013
Fasting glucose (mg/dL)	100.5 (91.0–117.8)	102.5 (93.5–134.3)	0.181
Blood urea nitrogen (mg/dL)	20.1 (15.4–37.0)	16.3 (13.4–20.5)	0.002
Serum creatinine (mg/dL)	1.0 (0.7–2.3)	0.7 (0.5–0.9)	0.003
Serum total protein (g/dL)	6.8 (6.2–7.3)	6.9 (5.8–7.1)	0.522
Serum albumin (g/dL)	3.7 (3.2–4.2)	3.9 (3.0–4.5)	0.007
ESR (mm/h)	67.0 (28.8–107.0)	39.5 (12.0–105.5)	<0.001
CRP (mg/L)	13.6 (2.2–63.9)	1.8 (0.7–80.9)	0.001
During follow-up			
Advanced systemic complications			
ACM (N, (%))	39 (16.3)	8 (25.0)	0.223
Follow-up duration based on ACM (months)	52.6 (22.3–85.3)	69.6 (5.9–107.9)	0.007
Relapse (N, (%))	66 (27.6)	7 (21.9)	0.492
Follow-up duration based on relapse (months)	33.9 (11.9–67.5)	37.7 (5.9–102.2)	0.016
ESKD (N, (%))	50 (20.9)	2 (6.3)	0.048
Follow-up duration based on ESKD (months)	41.3 (13.6–77.4)	69.6 (5.9–107.9)	0.001
CVA (N, (%))	16 (6.7)	2 (6.3)	>0.999
Follow-up duration based on CVA (months)	48.7 (18.7–83.9)	69.6 (5.9–107.9)	0.003
ACS (N, (%))	6 (2.5)	1 (3.1)	0.589
Follow-up duration based on ACS (months)	51.8 (21.9–84.5)	69.6 (5.9–107.9)	0.005
Medications administered (N, (%))			
Glucocorticoid	231 (96.7)	31 (96.9)	>0.999
Rituximab	60 (25.1)	3 (9.4)	0.048
Cyclophosphamide	131 (54.8)	12 (37.5)	0.065
Mycophenolate mofetil	74 (31.0)	3 (9.4)	0.011
Azathioprine	112 (46.9)	14 (43.8)	0.740
Tacrolimus	19 (7.9)	4 (12.5)	0.329
Methotrexate	32 (13.4)	12 (37.5)	<0.001

Values are expressed as a median (25–75 percentile) or N (%). ANCA: antineutrophil cytoplasmic antibody; MPA: microscopic polyangiitis; GPA: granulomatosis with polyangiitis; BMI: body mass index; T2DM: type 2 diabetes mellitus; MPO: myeloperoxidase; P: perinuclear; PR3: proteinase 3; C: cytoplasmic; BVAS: the Birmingham Vasculitis Activity Score; FFS: the Five-Factor Score; SF-36: 36-item Short-Form Survey; PCS: Physical Component Summary; MCS: Mental Component Summary; CNS: central nervous system; PNS: peripheral nervous system; ESR: erythrocyte sedimentation rate; CRP: C-reactive protein; ACM: all-cause mortality; ESKD: end-stage kidney disease; CVA: cerebrovascular accident; ACS: acute coronary syndrome.

**Table 4:** Multivariable Cox proportional hazards analysis for subsequent end-stage kidney disease in patients with MPA or GPA.

Variables	HR	95% CI	*p*-Value
Age, per 1-year increase	1.035	1.008–1.062	0.009
Female sex (vs. male sex)	1.247	0.671–2.316	0.485
GPA (vs. MPA)	0.970	0.510–1.844	0.926
Serum creatinine, per 1 mg/dL increase	1.589	1.453–1.738	<0.001
Any ANCA positivity (vs. negativity)	2.764	0.651–11.740	0.168

MPA: microscopic polyangiitis; GPA: granulomatosis with polyangiitis; ANCA: antineutrophil cytoplasmic antibody; HR: hazard ratio; CI: confidence interval.

## Data Availability

The datasets collected and/or analysed in the present study are available upon request from the corresponding author.

## Supplementary Materials

The following supporting information can be downloaded at https://www.mdpi.com/article/10.3390/medicina62091699/s1, Figure S1: Comparison of cumulative patients’ and ESKD-free survival rates among patients without renal insufficiency.

## Abbreviations

The following abbreviations are used in this manuscript:

MPA	microscopic polyangiitis
GPA	granulomatosis with polyangiitis
ANCA	antineutrophil cytoplasmic antibody
AAV	ANCA-associated vasculitis
MPO	myeloperoxidase
PR3	proteinase 3
EMA	European Medicines Agency
ACR	American College of Rheumatology
EULAR	European Alliance of Associations for Rheumatology
IRB	Institutional Review Board
BVAS	Birmingham Vasculitis Activity Score
FFS	Five-Factor Score
SF-36	36-item Short-Form Survey
PCS	Physical Component Summary
MCS	Mental Component Summary
ESR	erythrocyte sedimentation rate
CRP	C-reactive protein
ACM	all-cause mortality
ESKD	end-stage kidney disease
CVA	cerebrovascular accident
ACS	acute coronary syndrome
P-ANCA	perinuclear antineutrophil cytoplasmic antibody
C-ANCA	cytoplasmic antineutrophil cytoplasmic antibody
BMI	body mass index
T2DM	type 2 diabetes mellitus
CNS	central nervous system
PNS	peripheral nervous system
HR	hazard ratio
CI	confidence interval

## References

[1] Jennette J.C., Falk R.J., Bacon P.A., Basu N., Cid M.C., Ferrario F., Flores-Suarez L.F., Gross W.L., Guillevin L., Hagen E.C. (2013). 2012 revised International Chapel Hill Consensus Conference Nomenclature of Vasculitides. Arthritis Rheum..

[2] Watts R., Lane S., Hanslik T., Hauser T., Hellmich B., Koldingsnes W., Mahr A., Segelmark M., Cohen-Tervaert J.W., Scott D. (2007). Development and validation of a consensus methodology for the classification of the ANCA-associated vasculitides and polyarteritis nodosa for epidemiological studies. Ann. Rheum. Dis..

[3] Hakroush S., Tampe D., Ströbel P., Korsten P., Tampe B. (2021). Comparative Histological Subtyping of Immune Cell Infiltrates in MPO-ANCA and PR3-ANCA Glomerulonephritis. Front. Immunol..

[4] Arnold S., Kitching A.R., Witko-Sarsat V., Wiech T., Specks U., Klapa S., Comdühr S., Stähle A., Müller A., Lamprecht P. (2024). Myeloperoxidase-specific antineutrophil cytoplasmic antibody-associated vasculitis. Lancet Rheumatol..

[5] Falde S.D., Fussner L.A., Tazelaar H.D., O’Brien E.K., Lamprecht P., Konig M.F., Specks U. (2024). Proteinase 3-specific antineutrophil cytoplasmic antibody-associated vasculitis. Lancet Rheumatol..

[6] Suppiah R., Robson J.C., Grayson P.C., Ponte C., Craven A., Khalid S., Judge A., Hutchings A., Merkel P.A., Luqmani R.A. (2022). 2022 American College of Rheumatology/European Alliance of Associations for Rheumatology classification criteria for microscopic polyangiitis. Ann. Rheum. Dis..

[7] Robson J.C., Grayson P.C., Ponte C., Suppiah R., Craven A., Judge A., Khalid S., Hutchings A., Watts R.A., Merkel P.A. (2022). 2022 American College of Rheumatology/European Alliance of Associations for Rheumatology classification criteria for granulomatosis with polyangiitis. Ann. Rheum. Dis..

[8] Pyo J.Y., Lee L.E., Park Y.B., Lee S.W. (2023). Comparison of the 2022 ACR/EULAR Classification Criteria for Antineutrophil Cytoplasmic Antibody-Associated Vasculitis with Previous Criteria. Yonsei Med. J..

[9] Choi C.B., Park Y.B., Lee S.W. (2019). Antineutrophil Cytoplasmic Antibody-Associated Vasculitis in Korea: A Narrative Review. Yonsei Med. J..

[10] Kitching A.R., Anders H.J., Basu N., Brouwer E., Gordon J., Jayne D.R., Kullman J., Lyons P.A., Merkel P.A., Savage C.O. (2020). ANCA-associated vasculitis. Nat. Rev. Dis. Primers.

[11] Bossuyt X., Cohen Tervaert J.W., Arimura Y., Blockmans D., Flores-Suárez L.F., Guillevin L., Hellmich B., Jayne D., Jennette J.C., Kallenberg C.G. (2017). Position paper: Revised 2017 international consensus on testing of ANCAs in granulomatosis with polyangiitis and microscopic polyangiitis. Nat. Rev. Rheumatol..

[12] McAdoo S.P., Medjeral-Thomas N., Gopaluni S., Tanna A., Mansfield N., Galliford J., Griffith M., Levy J., Cairns T.D., Jayne D. (2019). Long-term follow-up of a combined rituximab and cyclophosphamide regimen in renal anti-neutrophil cytoplasm antibody-associated vasculitis. Nephrol. Dial. Transplant..

[13] Phatak S., Aggarwal A., Agarwal V., Lawrence A., Misra R. (2017). Antineutrophil cytoplasmic antibody (ANCA) testing: Audit from a clinical immunology laboratory. Int. J. Rheum. Dis..

[14] Moiseev S., Cohen Tervaert J.W., Arimura Y., Bogdanos D.P., Csernok E., Damoiseaux J., Ferrante M., Flores-Suárez L.F., Fritzler M.J., Invernizzi P. (2020). 2020 international consensus on ANCA testing beyond systemic vasculitis. Autoimmun. Rev..

[15] Chung S.A., Langford C.A., Maz M., Abril A., Gorelik M., Guyatt G., Archer A.M., Conn D.L., Full K.A., Grayson P.C. (2021). 2021 American College of Rheumatology/Vasculitis Foundation Guideline for the Management of Antineutrophil Cytoplasmic Antibody-Associated Vasculitis. Arthritis Rheumatol..

[16] Hellmich B., Sanchez-Alamo B., Schirmer J.H., Berti A., Blockmans D., Cid M.C., Holle J.U., Hollinger N., Karadag O., Kronbichler A. (2024). EULAR recommendations for the management of ANCA-associated vasculitis: 2022 update. Ann. Rheum. Dis..

[17] Murray C.J., Atkinson C., Bhalla K., Birbeck G., Burstein R., Chou D., Dellavalle R., Danaei G., Ezzati M., Fahimi A. (2013). The state of US health, 1990–2010: Burden of diseases, injuries, and risk factors. JAMA.

[18] Mukhtyar C., Lee R., Brown D., Carruthers D., Dasgupta B., Dubey S., Flossmann O., Hall C., Hollywood J., Jayne D. (2009). Modification and validation of the Birmingham Vasculitis Activity Score (version 3). Ann. Rheum. Dis..

[19] Guillevin L., Pagnoux C., Seror R., Mahr A., Mouthon L., Toumelin P.L. (2011). French Vasculitis Study Group. The Five-Factor Score revisited: Assessment of prognoses of systemic necrotizing vasculitides based on the French Vasculitis Study Group (FVSG) cohort. Medicine.

[20] Han C.W., Lee E.J., Iwaya T., Kataoka H., Kohzuki M. (2004). Development of the Korean version of Short-Form 36-Item Health Survey: Health related QOL of healthy elderly people and elderly patients in Korea. Tohoku J. Exp. Med..

[21] Mantovani A., Garlanda C. (2023). Humoral Innate Immunity and Acute-Phase Proteins. N. Engl. J. Med..

[22] Webster A.C., Nagler E.V., Morton R.L., Masson P. (2017). Chronic Kidney Disease. Lancet.

[23] Hankey G.J. (2017). Stroke. Lancet.

[24] Kotecha T., Rakhit R.D. (2016). Acute coronary syndromes. Clin. Med..

[25] Lionaki S., Hogan S.L., Jennette C.E., Hu Y., Hamra J.B., Jennette J.C., Falk R.J., Nachman P.H. (2009). The clinical course of ANCA small-vessel vasculitis on chronic dialysis. Kidney Int..

[26] Scurt F.G., Bose K., Hammoud B., Brandt S., Bernhardt A., Gross C., Mertens P.R., Chatzikyrkou C. (2022). Old known and possible new biomarkers of ANCA-associated vasculitis. J. Autoimmun..

[27] Zhang P., Yan S.J., Hu J., Liu H.P., Xia W., Yang M., Kuang Q.-H., Shi K.-L., Fu M.-Z., Gao C.-L. (2024). Clinical outcomes and clinico-pathological correlations in children with MPO-ANCA-associated glomerulonephritis showing renal arteritis. J. Investig. Med..

[28] Sachez-Alamo B., Moi L., Bajema I., Berden A., Flossmann O., Hruskova Z., Jayne D., Wester-Trejo M., Wallquist C., Westman K. (2024). Long-term outcome of kidney function in patients with ANCA-associated vasculitis. Nephrol. Dial. Transplant..

[29] Ahn S.S. (2024). Renal prognosis in patients with new-onset microscopic polyangiitis and granulomatosis with polyangiitis requiring dialysis and potential predictor of renal function recovery. J. Rheum. Dis..

