# Supplementary material for: Association Between Any Circulating ANCA Positivity at Diagnosis and Subsequent End-Stage Kidney Disease in Microscopic Polyangiitis and Granulomatosis with Polyangiitis
Source: Medicina (Kaunas). 2026 Sep 4;62(9):1699. doi: 10.3390/medicina62091699 (PMC13609817; doi:10.3390/medicina62091699)
Supplement: Supplementary file 1 [file medicina-62-01699-s001.zip › medicina-4473922-supplementary.pdf]

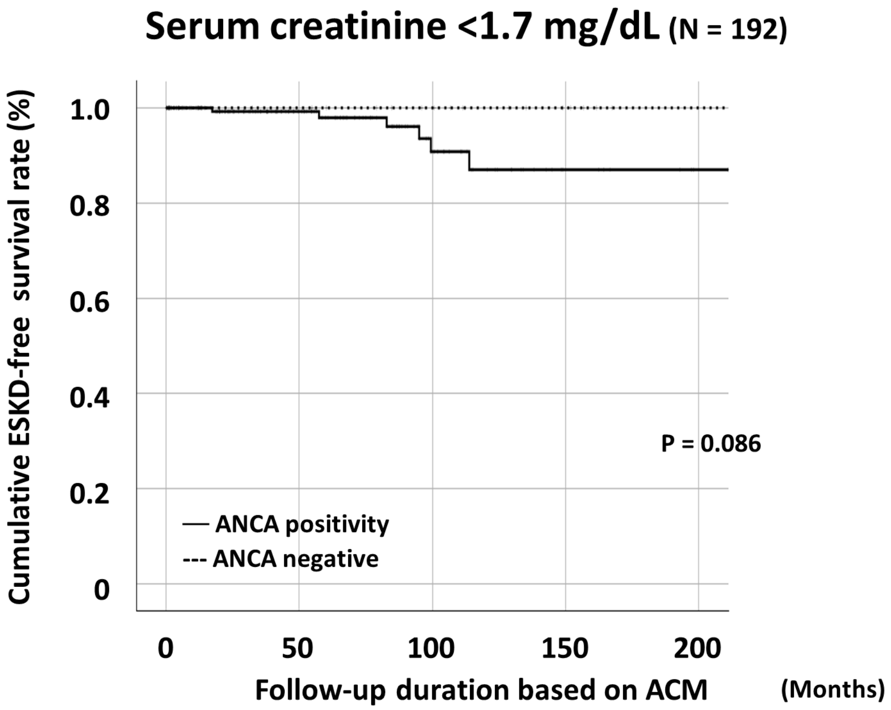

Figure S1. Comparison of cumulative patients' and ESKD-free survival rates among patients without renal insufficiency.
